# Challenges in Chagas Disease Drug Development

**DOI:** 10.3390/molecules25122799

**Published:** 2020-06-17

**Authors:** Amanda F. Francisco, Shiromani Jayawardhana, Francisco Olmo, Michael D. Lewis, Shane R. Wilkinson, Martin C. Taylor, John M. Kelly

**Affiliations:** 1 Department of Infection Biology, London School of Hygiene and Tropical Medicine Keppel Street, London WC1E 7HT, UK; amanda.francisco@lshtm.ac.uk (A.F.F.); shiromani.jayawardhana@lshtm.ac.uk (S.J.); francisco.olmo@lshtm.ac.uk (F.O.); Michael.lewis@lshtm.ac.uk (M.D.L.); martin.taylor@lshtm.ac.uk (M.C.T.); 2 School of Biological and Chemical Sciences, Queen Mary University of London Mile End Road, London E1 4NS, UK; s.r.wilkinson@qmul.ac.uk

**Keywords:** *Trypanosoma cruzi*, Chagas disease, drug development

## Abstract

The protozoan parasite *Trypanosoma cruzi* causes Chagas disease, an important public health problem throughout Latin America. Current therapeutic options are characterised by limited efficacy, long treatment regimens and frequent toxic side-effects. Advances in this area have been compromised by gaps in our knowledge of disease pathogenesis, parasite biology and drug activity. Nevertheless, several factors have come together to create a more optimistic scenario. Drug-based research has become more systematic, with increased collaborations between the academic and commercial sectors, often within the framework of not-for-profit consortia. High-throughput screening of compound libraries is being widely applied, and new technical advances are helping to streamline the drug development pipeline. In addition, drug repurposing and optimisation of current treatment regimens, informed by laboratory research, are providing a basis for new clinical trials. Here, we will provide an overview of the current status of Chagas disease drug development, highlight those areas where progress can be expected, and describe how fundamental research is helping to underpin the process.

## 1. Introduction

Chagas disease is endemic in many areas of Latin America, and is a particular problem amongst the rural poor, where an estimated 6–7 million people are infected with the causative agent, the protozoan parasite *Trypanosoma cruzi* [1]. The disease has also become a global health problem, with several hundred thousand infected individuals within migrant populations, mainly in the USA and Europe [2,3]. The principal route of *T. cruzi* infection is via hematophagous triatomine bugs, although oral (contaminated food and drink) and congenital transmission are also important, along with blood transfusion and organ transplantation. Encouragingly, public health measures, specifically insecticide spraying of poor-quality housing, have had a significant impact in breaking transmission cycles in some areas [4,5], but the infection is a zoonosis, and these measures will have to be sustained to maintain this improved situation. Eradication by this route is unlikely to be feasible. There are no vaccines against Chagas disease, and given the immunological complexity and long-term nature of the infection, progress in this area is uncertain. Therefore, new drugs, which avoid the drawbacks associated with the current therapeutic options, will have the potential to play a significant role in eliminating the massive disease burden that results from this infection, and in reducing the resulting constraints on the socio-economic development of many rural communities. The economic impact associated with Chagas disease is more than $7 billion per annum, a figure exceeding the total global costs linked to uterine, cervical and oral cancers [6,7]. For further comparison, the estimated productivity gains from elimination of lymphatic filariasis, schistosomiasis and soil-transmitted helminths from the Americas would amount to approximately $0.5 billion per annum [8].

*T. cruzi* is an obligate intracellular parasite with an extremely wide mammalian host range, and an ability to infect the vast majority of nucleated cells. Typically, transmission occurs when infected faeces from the insect vector are deposited near the bite wound after a blood-meal. Flagellated metacyclic trypomastigote forms of the parasite are then introduced via the wound or mucous membranes, by scratching or rubbing. Following host cell invasion, trypomastigotes escape from the parasitophorous vacuole into the cytoplasm, differentiate into the small rounded amastigote form, and replicate by binary fission. Four to five days later, after several rounds of division, and differentiation into bloodstream trypomastigotes, lysis of the host cell results in parasite release and dissemination of the infection.

In humans, the initial acute stage of the disease lasts 4–6 weeks, and is associated with patent parasitemia and infection of most tissues and organs. However, symptoms are usually mild and non-specific, with transient fever and muscle pain; the majority of individuals are unaware that they have been infected. In some cases, the disease can be more serious, particularly in children, where death can result from myocarditis or encephalopathy. Control of the infection is mediated predominantly by a strong CD8 + T cell response, which reduces the parasite burden by 2–3 orders of magnitude [9,10], although sterile immunity is rarely achieved. The disease transitions to an asymptomatic chronic stage, where the parasite burden is low and focal. Despite the life-long nature of the infection, the majority of individuals do not develop overt pathology, although a significant minority (~30%) progress to a symptomatic chronic state characterised by progressive cardiac and/or digestive disease. In most cases, this takes decades to become apparent. Cardiomyopathy is the most serious outcome of *T. cruzi* infection [11,12], and in many areas of South America it is a major cause of heart disease. The digestive symptoms, which include megaoesophagus and megacolon, also have serious consequences and can require surgery to alleviate the symptoms [13].

## 2. The Current Status of Chagas Disease Chemotherapy 

For the last 50 years, the orally-administered compounds benznidazole and nifurtimox have remained the only drugs available to treat *T. cruzi* infections [14,15]. However, they require long-term periods of administration (typically 60 days), are often noncurative (generally in the range 10–30% of cases), toxicity is a significant problem, and use during pregnancy is contraindicated [16,17,18]. Side effects are reported in up to 90% of patients, with cutaneous, digestive and neurological complications being the most common [19]. As a result, patient compliance can be a major issue. Furthermore, because only a minority of cases are diagnosed in the acute or asymptomatic chronic stages, the number of *T. cruzi*-infected individuals offered antiparasitic drug treatment is relatively small [20,21].

**Figure 1:**
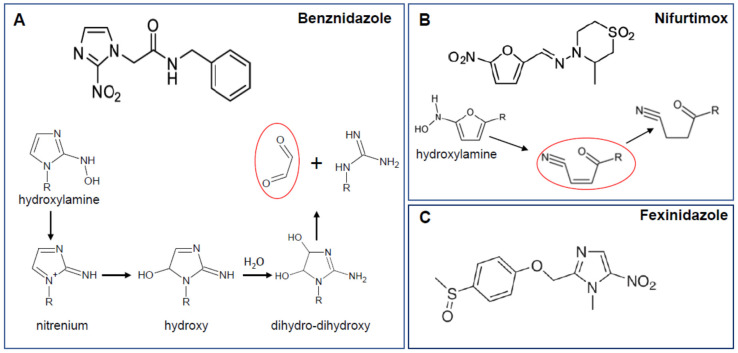
Nitroaromatic drugs used to treat *Trypanosoma cruzi* infections, or undergoing clinical trial. (**A**) Reductive metabolism of benznidazole, initiated by TcNTR-1, leads to the production of an unstable hydroxylamine derivative. This is readily converted to a hydroxy intermediate (possibly through a nitrenium ion form), which then reacts with water to generate a dihydro-dihydrooxy. This slowly breaks down to release the highly reactive dialdehyde, glyoxal (circled in red) [24]. The intermediaries and final product can form adducts with proteins, DNA, and small molecules such as glutathione and trypanothione. (**B**) Nifurtimox is reduced by TcNTR-1, leading to the generation of an unstable hydroxylamine. This decomposes, potentially via a ketoxime intermediate, to form unsaturated (circled in red) and then saturated open-chain nitriles [25]. The unsaturated form mediates trypanocidal activity. (**C**) Fexinidazole, an NTR-1-activated 5-nitroimidazole prodrug [31], has recently been approved as an oral treatment for African trypanosomiasis [32,33]. It outperforms other nitroaromatic drugs as a curative treatment for experimental *T. cruzi* infections [34], and is undergoing clinical trial against Chagas disease [35].

Benznidazole and nifurtimox are nitroheterocyclic compounds, containing a nitro group attached to imidazole and furan rings, respectively (Figure 1A,B). They function as prodrugs and are bioactivated within the parasite by the same mitochondrial-localised flavin-dependent enzyme, the type 1 nitroreductase TcNTR-1 [22,23]. It is the substrate-specificity of TcNTR-1 and the lack of a corresponding enzyme in the mammalian host that accounts for the selectivity of drug action. Reductive drug metabolism generates a series of reactive intermediates that have trypanocidal activity, most notably glyoxal in the case of benznidazole [24], and an unsaturated open-chain nitrile with nifurtimox [25] (Figure 1A,B). For benznidazole, drug-induced mutagenesis has been identified as a possible mode of action, resulting, for example, in disruption to DNA-repair mechanisms, and chromosome instability [26,27,28,29,30]. Possible inducers of mutagenesis include reactive drug metabolites, enhanced oxidative stress resulting from drug adduct interactions with trypanothione, and the production of 8-oxo-guanine and other oxidised nucleotides.

Cross-resistance to both benznidazole and nifurtimox can be readily generated *in vitro*, often resulting from reduced expression of functional TcNTR-1 that impacts on the ability of the parasite to reduce nitro-drugs [22,36,37]. Given the relatively small numbers of patients who are actually drug-treated [20,21], the infrequency with which genetic exchange occurs in *T. cruzi*, and the extensive animal reservoir of the parasite, it is unlikely that acquired drug-resistance will develop or spread widely at a population level. However, natural parasite populations do display a broad range of susceptibility to benznidazole [36,38,39], a factor that could have a role in some treatment failures. The level of sensitivity in natural strains is not obviously associated with specific parasite lineages, and not linked to polymorphisms in the TcNTR-1 gene, suggesting that other processes could be involved. These might include alternative drug-activation enzymes [40], enhancement of oxidative defence [41] or DNA repair pathways [29], increased drug efflux [42] or decreased drug uptake. In addition, it has been observed that within infected cells, small numbers of the amastigote population can enter a state of apparent dormancy in which they display increased drug tolerance [43]. In the absence of new treatments, it is important that these alternative mechanisms are explored further to optimise the usage of the current drugs.

Chronic chagasic heart disease is characterised by inflammation, fibrosis, blood clots and arrhythmias, which lead to progressive cardiac failure, and in some cases sudden death [44,45]. It is now generally accepted that the presence of the parasite is a prerequisite for driving the development of this pathology [46,47], although persistent infection of the target organs may not be essential. Rather, cumulative collateral damage could result from localised inflammatory immune responses generated against continuous rounds of reinfection by parasites trafficked from other more immunotolerant sites of persistence. Importantly, the central role played by the parasite in disease pathogenesis strongly implies that effective therapy should block or reduce the development of pathology. Evidence supporting this has come from several reports on the beneficial effects of curative treatment of acute stage infections in experimental models [48,49,50,51,52]. Studies, using highly sensitive *in vivo* imaging, further suggest that the beneficial outcomes, in terms of protection against cardiac pathology, may be lessened if treatment is withheld until chronic stage symptoms develop [53]. In humans, the evidence suggests that curative treatment of acute-stage infections also provides long-term therapeutic efficacy [54], with a gradual diminution of benefit if treatment is delayed. Much of the cardiac damage in chronic patients seems to be irreversible once it has developed. For example, in the BENEFIT study, no significant improvements in cardiac function were observed in chronic chagasic patients 5 years after benznidazole treatment [55]. In this clinical trial, volunteers had been preselected on the basis that they already displayed cardiac disease, a decision that has provoked some discussion [56,57,58]. Taken together, the current evidence supports the premise that early curative treatment of *T. cruzi* infections will have optimal benefit in preventing the development of symptomatic cardiac pathology and other outcomes.

More recently, the BENDITA phase II clinical trial, carried out in Bolivia under the auspices of the Drugs for Neglected Diseases initiative (DND*i*), has investigated the impact of reducing the benznidazole treatment period from 8 weeks to 2 weeks, and the dose from 300 mg/day to 150 mg [59]. In both cases, the curative rate (~80%) was similar to that in those who received the standard regimen, and was accompanied by a reduction in the number of patients who discontinued treatment because of drug toxicity. Although these findings are preliminary, they highlight the potential for minimising the side effects of benznidazole, maintaining antiparasitic efficacy and removing some of the barriers to successful treatment.

## 3. Progress in Chagas Disease Drug Development

Both target-based and phenotypic screening approaches have been widely applied to Chagas disease drug-discovery. In the case of the former, there are an increasing number of enzymes and metabolic pathways that have been genetically and/or chemically validated, and these have been the focus of much research. For example, in *T. cruzi*, as in fungi, ergosterol is a major and essential component of cell membranes, and inhibitors of its biosynthetic pathway have been prime candidates for drug development [60,61]. Azoles, which act through inhibition of lanosterol 14-α demethylase (CYP51), are effective antifungal agents [62], and attempts to repurpose several of these for use against *T. cruzi* infections have generated considerable interest. Most prominently, the antifungal drug posaconazole, a highly efficient inhibitor *T. cruzi* CYP51, showed great promise in preliminary studies and was advanced into clinical trial. However, it was found to have limited curative potential as a monotherapy, and provided no added benefit when used in combination with benznidazole [63,64]. Similarly, the related triazole ravuconazole also performed sub-optimally in clinical trial [65]. These failures were a great disappointment to the Chagas disease research community. More recent studies have demonstrated that although posaconazole has an *in vitro* EC50 in the low nanomolar range, a subpopulation of parasites seem refractory to drug activity, a phenomenon that shows great variability between strains [66]. Furthermore, *in vivo* experiments have revealed that posaconazole has limited ability to confer sterile cure on murine models, despite an initial pronounced knockdown in the parasite burden [67,68]. Understanding why cytostatic drugs like posanconazole struggle to eliminate *T. cruzi* infections will be critical to inform the drug development process. An unexpectedly high number of “hits” obtained from phenotypic screens have been found to be inhibitors of CYP51, perhaps reflecting features of the active site of the enzyme [69]. Given the failure of posaconazole in clinical trials and a desire to focus on alternative targets, small molecule ligands with CYP51 inhibitory properties are now actively excluded from further progress along drug development pipelines [70].

Proteases have been successful drug targets in several pathogens, and members of the cruzipain family of *T. cruzi* cysteine proteases have been intensively studied in this context [71,72]. Cruzipain functions in a wide variety of roles throughout the parasite life-cycle, including host cell invasion, differentiation, evasion of the immune response, and in several aspects of host–parasite interaction [73,74,75,76]. The enzyme family has been subject to intense biochemical and structural scrutiny, with inhibitors from several chemical classes having been shown to have potent trypanocidal properties and promising *in vivo* activity, with treatment outcomes that include parasitological cure and reduction of cardiac pathology [77,78,79]. Although none of these have yet progressed to clinical trial, parasite cysteine proteases remain a research area with some promise. The parasite oxidative defence pathway has also been an important focus of drug-related research. Many components and pathways display parasite-specific features and have been genetically validated [80,81]. For example, the unique thiol trypanothione (a glutathione:spermidine conjugate) plays a central role in maintaining redox balance within the parasite [82,83,84]. The key enzyme trypanothione reductase (TR) has been widely targeted for drug design, a process that has benefitted considerably from the availability of a crystal structure [85]. Other potential drug targets that have been investigated in *T. cruzi* and related parasites include methionyl-tRNA synthetase [86] and enzymes involved in glycoconjugate biosynthesis [87].

**Figure 2:**
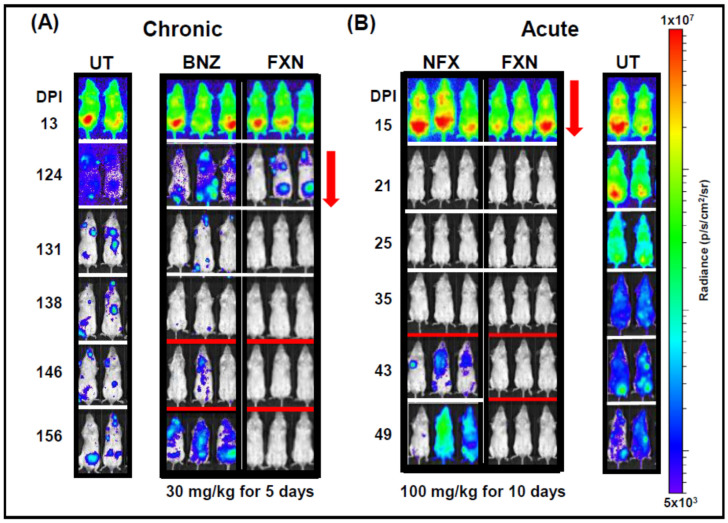
Fexinidazole outperforms benznidazole and nifurtimox as a treatment for experimental *Trypanosoma cruzi* infections. (**A**) BALB/c mice were infected with bioluminescent *T. cruzi* CL Brener strain [99]. At the chronic stage of infection (124 days), they were treated with benznidazole (BNZ) or fexinidazole (FXN) (30 mg/kg, orally, once daily) for 5 days (marked by red arrow). Treated mice were immunosuppressed on days 138, 142 and 146 using cyclophosphamide (200 mg/kg, i.p) (red lines). Images were acquired using the Lumina II IVIS system (Caliper Life Science) [100]. (**B**) BALB/c mice at the acute stage of infection (15 days) were treated with nifurtimox (NFX) or FXN (100 mg/kg, orally, once daily) for 10 days (red arrow), and then immunosuppressed on days 35, 39 and 43 using cyclophosphamide (red line). UT, untreated control mice. Heat-maps are on log10 scales and indicate intensity of bioluminescence from low (blue) to high (red). Full data set available in reference [34].

Despite the widespread application of rational drug design, few new compounds identified by this approach have yet advanced far along the Chagas disease drug development pipeline. This has renewed interest in exploring the potential of other nitroaromatics that might have superior properties to benznidazole and nifurtimox, even though they may share a common mechanism of bioactivation, mediated by TcNTR-1. The recent approval of fexinidazole (Figure 1C) by the European Medicines Agency as an oral treatment for African trypanosomiasis caused by *Trypanosoma brucei gambiense* [32,33] has further strengthened this interest. Fexinidazole, an NTR-1-activated 5-nitroimidazole pro-drug [31], has been shown to outperform both benznidazole and nifurtimox as a curative treatment for experimental *T. cruzi* infections [34] (Figure 2A,B, as example), and a clinical trial to assess efficacy and tolerability against patients with asymptomatic chronic infections has been undertaken [35]. In addition, there have also been a number of recent studies reporting encouraging preliminary data on the antiparasite activities of other novel nitroaromatics [88], and of a new generation of diverse organometallic compounds [89,90,91,92].

Probably the most prominent recent development in Chagas disease drug research has been the increased use of high-throughput phenotypic screening [93,94,95]. The requirement for large compound libraries, robotic sample handling equipment, expertise in parasite biology, and over-arching funding mechanisms has brought together both the academic and commercial sectors, with not-for-profit drug development agencies, such as the DND*i*. These consortia are international in make-up and encompass large research teams and networks with expertise in medicinal chemistry, pharmacology, toxicology, molecular biology, biochemistry, and clinical sciences. Examples of such collaborative partnerships include the GSK and Tres Cantos Open Lab Foundation; initiatives such as the kinetoplastid boxes, each with ~ 200 chemical hits, which have been made freely available to the research community [96]; and the Novartis-led project that identified the parasite proteasome inhibitor GNF6702, which has broad spectrum anti-kinetoplastid activity [97]. This breakdown of drug-development into its component parts has brought a more systematic approach to the process and has offered economies of scale that are particularly important when the research area is drugs for neglected diseases.

The failure of posaconazole in clinical trial identified the urgent need for improved preclinical methodologies with greater predictive power. One response has been the development of long-term washout experiments, which demonstrated that even with prolonged posaconazole exposure *in vitro* [98], there is an inability to kill all parasites. This is indicative of phenotypic heterogeneity within the population, and perhaps the presence of a metabolically quiescent sub-group that is more resistant to drug activity [43]. These findings further highlight where gaps in our understanding of parasite biology can impact negatively on drug development. For the remainder of this review, we will discuss how improvements in imaging procedures applied to predictive murine models have allowed some of these issues to be addressed, and have helped to streamline aspects of the drug screening process.

## 4. Highly Sensitive In Vivo Bioluminescence Imaging and Its Application to the Chagas Disease Drug Development Pipeline

Mice have been widely used as experimental models in Chagas disease drug research. However, during the chronic stage of infection, which is the main target for new therapeutics, the parasite burden is extremely low, the sites of infection are highly localised, and imaging suggests that these foci are often transient [100]. As a result, neither light microscopy nor PCR-based methodologies can be used to reliably monitor *in vivo* infections in real time, and even end-point assays can be uncertain [67]. However, it is now clear that non-invasive bioluminescence imaging of mice infected with *T. cruzi* that stably express a firefly luciferase gene can provide a method for following chronic infections, if sufficient sensitivity can be achieved [99,100,101]. The major determinants of sensitivity in these situations are the level of expression of the luciferase enzyme, and the wavelength of light emitted by oxidation of the luciferin substrate. Since this is an ATP-dependent reaction, only live parasites are detected, which is not necessarily the case with PCR. Insertion of a luciferase gene into highly expressed ribosomal loci of the parasite solved the first of these issues, and the use of a genetically engineered codon-optimised red-shifted luciferase [102] addressed the second. Visible light towards the red end of the spectrum (617 nm in this case) has greater tissue penetration due to reductions in the absorbance and scattering of the emitted light. Using these systems, chronic *T. cruzi* infections in mice can be monitored for more than one year, with a limit of detection close to 100 parasites using *in vivo* imaging [100], and less than 20 parasites when *ex vivo* imaging is subsequently used to examine mouse tissue [103]. With *in vivo* imaging, there is a linear relationship between the parasite burden (above 1000 parasites) and whole-body bioluminescence intensity [99]. In addition to a central role in *in vivo* drug screening (Figure 2), these imaging procedures have provided new insights into infection dynamics and tissue tropism [100,101,104], disease pathogenesis [53] and vaccine development [105].

**Figure 3:**
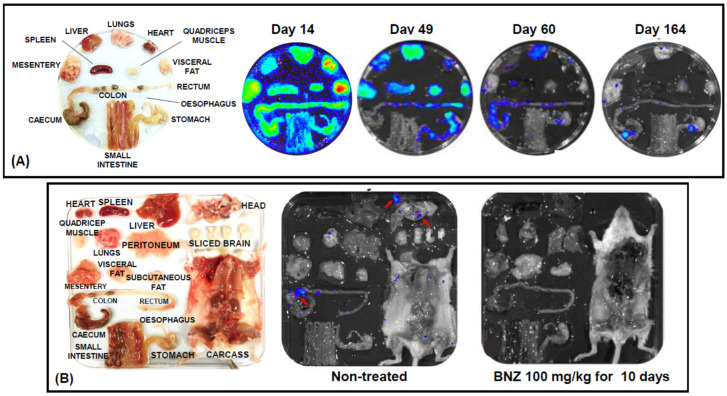
Monitoring parasite tropism and drug activity in chronic *Trypanosoma cruzi* infections using *ex vivo* imaging. (**A**) BALB/c mice were infected with the bioluminescent *T. cruzi* CL Brener strain. Animals were sacrificed at various points thereafter, and organs and tissue were removed, arranged in a Petri dish as indicated, and immersed in luciferin [100]. Bioluminescence imaging revealed wide dissemination and high parasite burden at the peak of the acute stage (14 days post-infection), and the effect of immune-mediated control of the infection during the transition to the chronic phase (typically day 40–60). In this infection model, the predominant long-term sites of parasite persistence are the colon and/or stomach. Infection of other organs/tissues is more sporadic, although parasites are often located in the skin [103]. (**B**) Exploiting *ex vivo* imaging to assess drug efficacy against chronic *T. cruzi* infection. Detailed information on parasite tropism and drug susceptibility can be established by including the entire carcass and head of the mouse in the imaging process. In the example shown, benznidazole (BZN) treatment has eliminated detectable parasites. In the nontreated mouse, infection of the head region was observed (parietal, frontal, zygomatic and lacrimal bones; red arrows).

The standard experimental model used for drug testing is the BALB/c mouse infected with the bioluminescent *T. cruzi* CL Brener strain, a parasite that belongs to the discrete typing unit (DTU) VI lineage. In terms of replication rate and virulence, the genetically modified strain is indistinguishable from the parental line [100]. The acute stage infection follows a regular profile in which the bioluminescence-inferred parasite burden reaches a peak at approximately 14 days post-infection (Figure 2B). At this time-point, it is brought under control by the adaptive immune system, resulting in a drop of up to 1000-fold in parasite numbers as the infection transitions to the chronic stage, approximately 50–60 days post-infection [100]. During the acute stage, parasites infect all organs and tissues, whereas in the chronic stage, they are mainly confined to the colon, stomach and skin (Figure 3A). In C3H/HeN mice, infection of skeletal muscle is also a regular feature of the chronic stage [101,103]. As in humans, *T. cruzi* infections are generally life-long, and *in vivo* imaging has revealed a highly dynamic profile during the chronic stage in which transient bioluminescence foci appear and disappear over a time period of hours. The precise nature of these foci is unknown, but they could represent infected phagocytes in the process of being trafficked from sites of persistence in the mouse to peripheral sites [47].

This bioluminescence imaging system has now been incorporated into several Chagas disease drug development programmes [34,52,106]. It can be used to monitor drug efficacy against both acute and chronic infections (Figure 2A,B, Figure 3B) [34,67], enables several different dosing regimens to be assessed in parallel, allows tissue-specific differences in drug sensitivity to be investigated, and can be adapted to study the correlation between drug activity and disease pathology [53]. Typically, when treatment is complete, the mice are followed for another 10–20 days to determine the extent of any relapse. They are then immunosuppressed by cyclophosphamide (3 injections, 200 mg/kg i.p., at 3-day intervals) to facilitate the expansion of any residual parasites to detectable levels (Figure 2A,B) [101]. As a final step, mice are designated as cured/non-cured on the basis of both *in vivo* and *ex vivo* imaging. The sensitivity of this system seems to circumvent many of the issues related to the high false-cure rate that can be a problem with PCR-based diagnosis, as well as providing valuable insights into the dynamics of drug activity. In addition, the ability to continuously monitor individual mice in a non-invasive manner provides more comprehensive data sets, and reduces the number of animals required for experimentation.

The crucial factor with any experimental model is the reliability with which findings are directly translatable to human patients. In mice, the infection profile revealed by bioluminescence imaging closely mirrors that in humans, with a clearly defined acute stage, that transitions to a life-long chronic infection characterised by an extremely low parasite burden (Figure 2) [100]. This general trend holds true in a range of mouse models, although there can be some minor variation in the precise timing of events [101], a situation that also seems to be the case in humans. In terms of drug efficacy, the bioluminescence mouse model was predictive of the failure of posaconazole to cure human infections, despite impressive transient reductions in the parasite load [67]. Similar murine experiments have also demonstrated that benznidazole and nifurtimox have greater curative efficacy in the chronic stage than in the acute stage [34]. This may simply reflect the much larger number of parasites that have to be eliminated during acute stage infections. If these findings can be extended to humans, it suggests that there could be scope to decrease the treatment length and/or the drug dose without reducing the chronic infection cure rate. As outlined above, preliminary data from the BENDITA clinical trial [59] suggest that this may be the case, a finding that could have an enormous impact on patient compliance. Likewise, an on-going clinical trial [35] will provide further information on predictive value of the bioluminescence model, which was used to demonstrate that fexinidazole has superior curative properties compared to the current front-line drugs [34]. Murine models coupled with bioluminescent parasites will also provide a flexible platform for exploring issues such as combination therapy, so that the relative compound doses can be rapidly optimised before being advanced into clinical trial [64,65].

## 5. Concluding Remarks

In recent years, technical advances in several areas of the drug development process have contributed to a more favourable landscape for improving the therapeutic options available to treat neglected tropical diseases. In parallel, changes in research practice, accompanied by a more sympathetic funding environment, have contributed to the establishment of large drug discovery consortia that take a more systematic and multidisciplinary approach. After many years in which few therapeutic candidates advanced far along the development pipeline, the outlook for improved treatments for Chagas disease is looking considerably more favourable.
